# Predictors of osteo-articular infection in *Staphylococcus aureus* bacteremia in a tertiary center between 2017 and 2022

**DOI:** 10.5194/jbji-11-5-2026

**Published:** 2026-01-09

**Authors:** Liselot Vandenbergen, Diego Castanares Zapatero, Sébastien Briol, Alexia Verroken, Leila Belkhir, Olivier Cornu, Jean Cyr Yombi

**Affiliations:** 1 Department of Internal Medicine and Infectious Diseases, Cliniques Universitaires Saint-Luc, UCLouvain, 1200 Brussels, Belgium; 2 Department of Intensive Care, Cliniques Universitaires Saint-Luc, UCLouvain, 1200 Brussels, Belgium; 3 Department of Microbiology, Cliniques Universitaires Saint-Luc, UCLouvain, 1200 Brussels, Belgium; 4 Department of Orthopaedic and Trauma Surgery, Cliniques Universitaires Saint-Luc, UCLouvain, 1200 Brussels, Belgium; 5 Neuromusculoskelatal Laboratory (NMSK), Institute of Experimental and Clinical Research (IREC), UCLouvain, 1200 Brussels, Belgium

## Abstract

**Introduction**: Among bloodstream infections, *Staphylococcus aureus *bacteremia (SAB) is associated with a particularly high mortality rate, which may be higher in patients with undiagnosed metastatic infections. The primary objective of the present study was to identify risk factors for osteo-articular infection (OAI) in patients with active SAB. **Methods:** A retrospective study was conducted in a single-center tertiary-care hospital in Brussels, Belgium. Data were collected on patients diagnosed with SAB between 2017 and 2022. **Results:** Among the 489 consecutive patients with SAB included in this study, 141 (28.8 %) had a concomitant osteo-articular infection (OAI), accounting for nearly one in three patients. These infections included osteomyelitis (12.7 %), native joint septic arthritis (NJSA) (8.8 %), spondylodiscitis (8.4 %), and prosthetic joint infection (PJI) (3.9 %). Univariate and multivariate analyses were performed to identify risk factors associated with OAI. The duration of bacteremia (OR (odds ratio): 1.27, 95 %, CI (confidence interval): 1.14–1.42, p<0.001 ) and community-acquired bacteremia (OR: 3.23, 95 % CI: 1.85–5.88, p<0.001 ) were associated with the occurrence of OAI. The presence of active cancer (OR: 0.14, 95 % CI: 0.06–0.31, p<0.001 ) and intensive care unit (ICU) admissions for SAB (OR: 0.31, 95 % CI: 0.17–0.56, p<0.001 ) were associated with a lower likelihood of OAI. **Conclusion:** In this cohort, OAI was very frequent during SAB and occurred in 28.8 %, particularly in patients with community-acquired SAB (CA-SAB) or those with a longer duration of bacteremia. These findings highlight the importance of a comprehensive diagnostic evaluation for both primary and secondary infection foci, such as OAI, in the setting of SAB.

## 1 Introduction

*Staphylococcus aureus* (SA) is a highly virulent pathogen responsible for severe, life-threatening infections such as sepsis, meningitis, endocarditis, and osteo-articular infection (OAI) (Edwards and Massey, 2011; Jin et al., 2021). Among bloodstream infections, *Staphylococcus aureus* bacteremia (SAB) carries a particularly high mortality, with 90 d mortality rates exceeding 25 % (Jin et al., 2021; Briol et al., 2024). After entering the bloodstream, SA frequently disseminates hematogenously, leading to metastatic foci in nearly 30 % of patients (Edwards and Massey, 2011; Kimmig et al., 2021). Due to its specific virulence mechanisms and the absence of a protective basement membrane in the synovium, SA demonstrates a marked tropism for joints (Jin et al., 2021;; Earwood et al., 2021; Garcia-Arias et al., 2011). Metastatic infections such as OAI frequently prolong the duration of treatment and hospitalization and worsen clinical outcomes (Kimmig et al., 2021). The incidence of OAI secondary to SAB has been reported to range from 12 % to 17 % (Jin et al., 2021).

Reported host-related risk factors for NJSA include advanced age, diabetes mellitus, immunosuppression, rheumatoid arthritis (RA), renal disease, intravenous drug use, hemarthrosis, pre-existing joint disease, and intra-articular corticosteroid injections (Jin et al., 2021; Kimmig et al., 2021; Earwood et al., 2021).

Risk factors for spondylodiscitis include advanced age, diabetes, malignancy, cirrhosis, immunosuppression, renal disease, intravenous drug use, and prior spinal surgery (Gouliouris et al., 2010; Placide and Reznicek, 2025). When secondary to SAB, the incidence ranges from 1.7 % to 3 % (Gouliouris et al., 2010).

Prosthetic joint infection (PJI) occurs in 1 %–2 % of primary arthroplasties (Patel, 2023), but the risk rises markedly in SAB, with incidences of 30 %–40 % among patients with an orthopedic prosthesis (Sendi et al., 2011). Reported risk factors include male sex, younger age, anemia, malnutrition, obesity, diabetes mellitus, cardiovascular disease, renal or hepatic dysfunction, immunosuppression, blood transfusion, prior PJI, intra-articular injections, intravenous drug use, and SAB (Patel, 2023).

### Study objectives

The primary objective of the present study was to identify variables associated with OAI in patients with active SAB.

A secondary objective was to compare patients with an orthopedic prosthesis who developed PJI during SAB to those who did not in order to identify clinical differences.

## 2 Material and methods

### 2.1 Study design

We conducted a retrospective single-center study at Cliniques Universitaires Saint Luc (CUSL), a tertiary hospital in Brussels, Belgium. All consecutive patients of ≥ 18 years who presented SAB and who were treated in our institution between January 2017 and December 2022 were included. The calendar year 2020 was excluded due to COVID-19-related disruptions, which compromised data completeness and case ascertainment, making them non-comparable with other years.

In this population with SAB, we searched for risk factors of concomitant OAI, including native joint septic arthritis (NJSA), osteomyelitis, PJI, and spondylodiscitis. Patients with diabetic foot infections were excluded from this study because of its different physiopathology.

### 2.2 Ethical issues

The institutional ethics committee granted a waiver of written informed consent for the analysis of retrospective patient data derived from routine clinical practice, in accordance with national and European regulations. Consequently, institutional ethical committee approval was granted for this study (no. CEHF 2024/05JUL/296).

### 2.3 Data collection and variable definition

Data were collected using our institutional medical records (Epic Systems, Verona, Wisconsin, USA). We reviewed patient files and collected the following data: demographics (age, sex); comorbidities (renal insufficiency, dialysis, cirrhosis, malignancies, use of immunosuppressive therapy, diabetes, heart failure, respiratory disease, vascular/valvular graft or orthopedic prosthesis); community- or hospital-acquired bacteremia (defined as bacteremia occurring more than 48 h after admission); symptoms (fever, chills, joint pain, back pain); diagnostic investigations (TTE – transthoracic echocardiography and TEE – transesophageal echocardiography, [18F] FDG PET/CT – positron emission tomography); supposed source of SAB; presence of septic arthritis or prosthetic joint infection and joint affected; length of hospitalization; intensive care unit (ICU) stay following SAB; duration of bacteremia (defined as the number of days with positive blood cultures); persistent bacteremia (defined as positive SA blood culture sampled from the patient at least 48 h after initiation of appropriate antibiotics); recurrence of bacteremia (defined as a relapse within 90 d), antimicrobial susceptibility testing (AST); type and duration of empirical and definitive treatment; and all-cause mortality at 2, 7, 90 d, and 1 year.

### 2.4 Statistical analysis

Continuous variables were summarized as means with corresponding standard deviations or medians with 25 and 75 percentiles, while categorical variables were described using counts and proportions. Group comparisons for continuous variables were carried out using the unpaired Student's t test or the Mann–Whitney U test, depending on the distribution of the data. For categorical variables, comparisons were made using either the $\chi^{2}$ test or Fisher's exact test, as appropriate. Associations between clinical variables and the presence of OAI in patients with SAB were assessed using logistic regression. Variables deemed to be clinically relevant were considered for inclusion.

A preliminary univariate analysis was performed, and predictors with a p value of less than 0.20 were entered into a multivariable model, which was constructed using a stepwise forward selection approach. Results were reported as odds ratios (ORs) with corresponding 95 % confidence intervals (CIs). All hypothesis testing was two-sided, and statistical significance was defined as p<0.05 . Analyses were performed using IBM SPSS Statistics, version 20.0 (IBM Corp., Armonk, NY, USA).

## 3 Results

### 3.1 Baseline characteristics

In total, 595 consecutive patients were notified as having SAB between 2017 and 2022, but 106 patients were excluded due to age ( < 18 years old) ( n= 83) or missing critical information ( n= 23).

In our cohort of 489 patients with SAB included in this study, the mean age was 66.5 ± 16.2 years, and 62.8 % of the patients were male. Among the patients, 31.1 % had diabetes mellitus, 28.6 % were immunosuppressed, and 24.7 % had an active cancer diagnosis.

The majority of infections (92 %) were caused by methicillin-susceptible *Staphylococcus aureus* (MSSA), while only 8 % were due to methicillin-resistant *Staphylococcus aureus* (MRSA). Hospital-acquired bacteremia accounted for 35.6 % of cases and was not associated with a higher proportion of MRSA strains.

In 20 % of cases, the origin of the SAB remained unidentified. The most frequently identified foci were soft tissue infection (18.8 %) and catheter-related blood stream infection (CRBSI) (13.9 %). Other common diagnoses included osteomyelitis (12.2 %), pneumonia (11 %), endocarditis (10.6 %), septic arthritis (8.8 %), and spondylodiscitis (8.4 %).

Among the 43 patients diagnosed with NJSA, the most commonly affected joint was the shoulder (18.6 %) followed by the hip (16.3 %), knee (14 %), and acromio-clavicular or sterno-clavicular joints (11.6 %). Information on exact localization was missing for two patients. A total of 10 patients had arthritis in multiple joints (23.2 %), but each patient was counted only once based on the first localization that was diagnosed. We noticed that, among the eight patients with shoulder NJSA, three patients had endocarditis and arthritis in other joints (37.5 %), three patients had CRBSI with septic phlebitis (37.5 %), and two patients had cellulitis of the upper limb (25 %).

Among the 19 patients diagnosed with PJI, the hip was involved in 13 cases (68.4 %), and the knee was involved in 6 (31.6 %). Notably, 32.8 % of patients who had an orthopedic prosthesis at the time of bacteremia developed a PJI. Baseline patient characteristics are summarized in Table 1.

**Table 1:** SAB main characteristics. All values in the table are presented as absolute frequencies and corresponding percentages, except for continuous variables, which are reported as means with standard deviations or medians and 25 and 75 percentiles (P25, P75).

Characteristics	Population
	SAB ( n= 489)
Age	
Mean – years (SD)	66.5 (16.2)
Distribution – no. (%)	
< 60 years	146 (29.9)
60–70 years	122 (24.9)
70–80 years	129 (26.4)
> 80 years	92 (18.8)
Gender – no. (%)	
Male	307 (62.8)
Female	182 (37.2)
Medical conditions – no. (%)	
Diabetes	152 (31.1)
Diabetic foot	45 (9.2)
Heart condition	169 (34.6)
Prosthetic valve	38 (7.8)
Heart device	42 (8.6)
Pulmonary disease	69 (14.1)
Cirrhosis	44 (9)
Renal insufficiency (GFR < 60 mL min^−1^)	232 (47.4)
Dialysis	52 (10.6)
Active cancer	121 (24.7)
Immunosuppression	140 (28.6)
Joint prosthesis	58 (11.9)
Knee prosthesis	30 (6.1)
Hip prosthesis	38 (7.8)
Shoulder prosthesis	3 (0.6)
Spinal arthrodesis	19 (3.9)
Symptoms – no. (%)	
Shivers	142 (29)
Fever > 38 °C	217 (44.4)
Arthralgia	55 (11.2)
Complementary exams	
TTE – no. (%)	347 (71) ( n= 347)
Sign of endocarditis	21 (6.1)
TEE – no. (%)	210 (43) ( n= 210)
Sign of endocarditis	41 (19.5)
[18F] FDG PET-CT – no. (%)	209 (42.7) ( n= 209)
Secondary foci	56 (26.8)
Mean delay of exam – days (SD)	10.6 (7.9)
Bacteremia	
Nosocomial bacteremia – no. (%)	174 (35.6)
Mean duration of bacteremia – days (SD)	2.4 (2.3)
Persistent bacteremia ( > 48H) – no. (%)	106 (21.7)

**Table 1:** Continued.

Characteristics	Population
	SAB ( n= 489)
Origin of infection – no. (%)	
Unknown	98 (20)
Soft tissue infection	97 (19.8)
CRBSI	68 (13.9)
Osteomyelitis	62 (12.7)
Pneumonia	54 (11)
Endocarditis	51 (10.4)
NJSA	43 (8.8) ( n= 43)
Shoulder	8 (18.6)
Hip	7 (16.3)
Knee	6 (14)
AC and SC joint	5 (11.6)
Ankle	5 (11.6)
Pelvis	4 (9.3)
Elbow	2 (4.7)
Foot	2 (4.7)
Hand	1 (2.3)
Wrist	1 (2.3)
Missing	2 (4.7)
Spondylodiscitis	41 (8.4)
Other	37 (7.6)
PJI	19 (3.9) ( n= 19)
Hip	13 (68.4)
Knee	6 (31.6)
Treatment	
Correct empirical treatment – no. (%)	332 (71.4)
Median duration of appropriate antibiotherapy – days (P25–P75)	20.0 (13–42)
Median duration of appropriate IV antibiotherapy – days (P25–P75)	15 (11–27)
Susceptibility testing – no. (%)	
MSSA	450 (92)
MRSA	39 (8)
Ciprofloxacin-R	43 (8.8)
Trimethoprim/sulfamethoxazole-R	17 (3.5)
Clindamycin-R	101 (20.7)
Erythromycin-R	142 (29)
Tetracyclin-R	6 (1.2)
Disease burden	
ICU after bacteremia – no. (%)	147 (30)
Median duration of hospitalization – days (P25–P75)	20 (12–34)
2 d mortality – no. (%)	18 (3.7)
7 d mortality – no. (%)	78 (16)
90 d mortality – no. (%)	190 (39.5)
1-year mortality – no. (%)	227 (47.3)
Recurrence of bacteremia at 90 d – no. (%)	29 (5.9)

Patients could have multiple foci or diagnoses; therefore, proportions do not sum to 100 %. SAB: Staphylococcus bacteremia. n : absolute number. SD: standard deviation. No. (%): number in percentage. GFR: glomerular filtration rate. TTE: transthoracic echocardiography. TTE: transesophageal echocardiography. [18F] FDG PET/CT: positron emission tomography. CRBSI: catheter related blood stream infection. NJSA: native joint septic arthritis. AC and SC: acromio-clavicular and sterno-clavicular. PJI: prosthetic joint infection. MRSA: methicillin-resistant *Staphylococcus aureus*. MSSA: methicillin-susceptible *Staphylococcus aureus*. R: resistant. ICU: intensive care unit.

### 3.2 Risk factors

Although 24 patients presented with multiple osteo-articular diagnoses simultaneously, each patient was counted only once in this analysis, giving a total of 141 patients with OAI (28.8 %). Univariate analysis identified several statistically significant risk factors for OAI: age (OR: 1.02, 95 % CI: 1–1.04, p= 0.005), duration of bacteremia (OR: 1.23, 95 % CI: 1.12–1.34, p<0.001 ), and persistent bacteremia (OR: 2.01, 95 % CI: 1.17–3.5, p= 0.011). Nosocomial bacteremia, active cancer, and ICU stay were inversely associated with the likelihood of OAI. Multivariate analysis confirmed that a longer duration of bacteremia (OR: 1.27, 95 % CI: 1.14–1.42, p<0.001 ) and community-acquired bacteremia (OR: 3.23, 95 % CI: 1.85–5.88, p<0.001 ) were significantly and independently associated with the occurrence of OAI. The presence of an active cancer diagnosis (OR: 0.14, 95 % CI: 0.06–0.31, p<0.001 ) and ICU stay (OR: 0.31, 95 % CI: 0.17–0.56, p<0.001 ) also remained protective factors against the development of OAI.

Univariate and multivariate analyses for risk factors associated with the subgroup NJSA and spondylodiscitis showed similar trends. However, ICU stay and cancer diagnosis were no longer significantly associated in the multivariate analysis. Univariate and multivariate analyses are displayed in Tables 2 and 3.

**Table 2:** Univariate analysis of risk factors for OAI (and subgroups NJSA/spondylodisicitis). All values in the table are presented as odds ratios (ORs) with their 95 % confidence interval (CI). /: non computable.

Risk	OAI	NJSA	Spondylodiscitis
factors	( n= 141)	( n= 43)	( n= 41)
OR 95 % CI[]			
Age	1.02 [1.01–1.03], p= 0.001	1.03 [1.01–1.05], p= 0.02	1.03 [1.01–1.05], p= 0.02
Male	1.66 [1.09–2.53], p= 0.019	0.90 [0.47–1.71], p= 0.74	1.48 [0.73–2.97], p= 0.27
Diabetes	1.13 [0.67–1.93], p= 0.640	0.74 [0.96–1.52], p= 0.42	0.51 [0.23–1.14], p= 0.10
Renal insufficiency	1.14 [0.77–1.69], p= 0.510	0.77 [0.41–1.44], p= 0.41	1.06 [0.58–2], p= 0.87
Cirrhosis	0.36 [0.15–0.87], p= 0.024	0.74 [0.22–2.5], p= 0.63	/
Cancer	0.24 [0.10–0.56], p= 0.001	0.37 [0.14–0.97], p= 0.04	0.31 [0.11–0.88], p= 0.03
Immunosuppression	0.49 [0.30–0.79], p= 0.003	1.09 [0.55–2.15], p= 0.81	0.49 [0.29–1.13], p= 0.09
Heart condition	1.01 [0.60–1.71], p= 0,960	1.02 [0.53–1.96], p= 0.96	1.10 [0.57–2.14], p= 0.78
Prosthetic valve	0.14 [0.02–1.06], p= 0.058	0.26 [0.03–1.97], p= 0.19	0.93 [0.27–3.17], p= 0.91
Pulmonary disease	0.84 [0.39–1.79], p= 0.60	0.60 [0.21–1.74], p= 0.35	0.48 [0.14–1.52], p= 0.20
Duration of bacteremia	1.23 [1.12–1.34], *p* < 0.001	1.18 [1.07–1.30], p= 0.001	1.36 [1.22–1.51], *p* < 0.001
Nosocomial bacteremia	0.16 [0.07–0.32], *p* < 0.001	0.27 [0.11–0.65], p= 0.004	0.23 [0.09–0.60], p= 0.002
Persisting bacteremia	2.07 [1.32–3.25], p= 0.002	2.6 [1.35–5], p= 0.004	5.57 [2.88–10.80], *p* < 0.001
ICU stay	0.47 [0.25–0.88], p= 0.019	0.59 [0.28–1.27], p= 0.18	1.09 [0.55–2.17], p= 0.80
MRSA	0.45 [0.14–1.53], p= 0.20	0.85 [0.25–0.90], p= 0.80	2.15 [0.84–5.50], p= 0.11

OAI: osteo-articular infection. NJSA: native joint septic arthritis. n : absolute number. p : probability value. ICU: intensive care unit. MRSA: methicillin-resistant *Staphylococcus aureus*.

**Table 3:** Multivariate analysis of risk factors for OAI (and subgroups NJSA/spondylodisicitis). All values in the table are presented as odds ratios (ORs) with their 95 % confidence interval (CI). Only predictors with a p value less than 0.20 were entered into the multivariable model. OAI: osteo-articular infection. NJSA: native joint septic arthritis. n : absolute number. ICU: intensive care unit.

Risk	OAI	NJSA	Spondylodiscitis	
factors	( n= 141)	( n= 43)	( n= 41)	
OR 95 % CI[]				
Male	1.80 [1.10–2.94], p= 0.019			
Cirrhosis	0.37 [0.14–0.99], p= 0.047			
Cancer	0.14 [0.06–0.31], p<0.001			
Duration of bacteremia	1.27 [1.14–1.42], p<0.001	1.15 [1.04–1.26], p= 0.007	1.32 [1.19–1.47], p<0.001	
Nosocomial bacteremia	0.31 [0.17–0.54], p<0.001	0.31 [0.13–0.78], p= 0.012	0.3 [0.11–0.80], p= 0.012	
ICU stay	0.31 [0.17–0.56], p<0.001			

### 3.3 Focus on prosthetic joint infection (PJI)

Of the 58 patients with an orthopedic prosthesis during SAB (11 % of the total cohort), 19 patients (32.8 %) were diagnosed with PJI. The mean age and general medical history were comparable between the PJI and non-PJI subgroups, except for a significantly higher prevalence of renal insufficiency in the non-PJI subgroup (59 % vs. 26.3 %, p= 0.02). The origin of bacteremia was more frequently nosocomial in the non-PJI subgroup compared to the PJI subgroup (25.6 % vs. 5.3 %, p= 0.06). There was no significant difference in clinical presentation such as chills or fever. However, all patients with PJI reported joint pain compared to only 25 % in the non-PJI subgroup ( p<0.001 ). Empirical antibiotic therapy was appropriate more often in the PJI subgroup (89.7 % vs. 60.7 %, p= 0.022), and the duration of treatment was significantly longer (74 d vs. 32 d, respectively). Among patients with PJI, 94.5 % underwent surgery.

No significant differences were observed in terms of hospital length of stay or ICU admission. The 90 d cumulative mortality in the PJI subgroup was 26.3 %, which was not statistically different from the non-PJI subgroup. However, 1-year mortality was significantly higher in the non-PJI subgroup (53.8 %) compared to the PJI subgroup (26.3 %, p= 0.044).

## 4 Discussion

In this retrospective study, we aimed to better characterize patients at risk of OAI while having active SAB. While previous literature reports the incidence of OAI secondary to SAB to range between 12 % and 17 % (Jin et al., 2021), our study found a notably higher incidence of 28.8 %. In the context of SAB, for native joint septic arthritis and spondylodiscitis, the reported incidences in the literature are estimated to be 7 % (van der Vaart et al., 2022) and 3 % (Gouliouris et al., 2010). In comparison, our study observed rates of 8.8 % for NJSA and 8.4% for spondylodiscitis. Regarding prosthetic joint infections, the literature describes an incidence between 30 % and 40 % (Sendi et al., 2011; Murdoch et al., 2001), aligning closely with our calculated rate of 32.8 %.

In our study, the main risk factors found to be significantly and independently associated with OAI during SAB were the duration of bacteremia (OR: 1.27, 95 % CI: 1.14–1.42, p<0.001 ) and CA-SAB (OR: 3.23, 95 % CI: 1.85–5.88, p<0.001 ). So, each additional day of bacteremia increased the odds of OAI by 27 %, and CA-SAB was associated with more than a 3-fold increase in odds of OAI. These findings are consistent with previous studies (Lambregts et al., 2020; Minejima et al., 2020; Horino et al., 2015). Both factors are also considered to be indicators of “complicated SAB”, defined by Fowler et al. (2003) and the Infectious Diseases Society of America (IDSA), which classifies complicated SAB as including any infection-related mortality, deep-seated infections (e.g., endocarditis, septic arthritis, embolic stroke), or relapse within 90 d. To aid clinicians in identifying patients with complicated SAB, a set of clinical risk factors has already been proposed: bacteremia for more than 48 h, fever persisting beyond 72 h, community acquisition of SAB, presence of prosthetic material, and skin findings suggestive of acute systemic infection. However, a recent 2024 study by van der Vaart et al. (2024) cautions against relying solely on clinical criteria to determine treatment duration, as initially recommended by IDSA guidelines. Their findings indicate that the IDSA definition of complicated SAB has a positive predictive value of 70.9 % and a negative predictive value of only 57.5 %, highlighting the potential for both over- and under-treatment with antibiotics. This raises the question of whether [18F] PET/CT imaging could play a beneficial role – either in all patients with SAB or selectively in high-risk cases. The literature on its mortality benefit and cost-effectiveness remains divided (Ong et al., 2023), and two randomized controlled trials are currently underway (TEPSTAR and PET-SAB) to provide more definitive answers (van der Vaart and Fowler, 2023). Our study was conducted in a large tertiary hospital in Belgium, where [18F] PET/CT imaging was readily available and thus used in 42 % of the total SAB population.

In our cohort, the cumulative 90 d mortality among patients with SAB was 39.5 %, consistently with findings from previous studies (Jin et al., 2021; Briol et al., 2024; Tong et al., 2025; van der Vaart et al., 2022). We did not compare mortality between patients who underwent [18F]FDG PET/CT and those who did not as this analysis was beyond the scope of the current study and has been previously addressed by our team (Briol et al., 2024).

Traditional risk factors for OAI were not found to be specifically associated with OAI in our cohort, likely due to their shared role as risk factors for SAB in general.

Interestingly, in our study, cancer appeared to be a protective factor against the development of OAI, although this effect was not observed in the smaller subgroups of spondylodiscitis or native septic arthritis. One hypothesis is that this patient population was less frequently screened with [18F] PET/CT during their episode of SAB (only 29.8 % underwent this imaging modality), whereas in the “osteomyelitis” group in particular, some infections were detected through [18F]PET/CT as metastatic foci. Another possible explanation is that neutropenia, common in cancer patients due to chemotherapy or hematologic malignancies, may reduce osteo-articular involvement due to the lack of circulating neutrophils, which seem essential for dissemination (Zhu et al., 2020), and, thus, fewer metastatic infections. This hypothesis has been studied previously, but no clear effect has been demonstrated. A prospective case control study, published in 2020, showed no difference in metastatic foci in neutropenic versus non-neutropenic patients (Ryu et al., 2020). Another study from 2023 by Camp et al. (2023) showed a potential protective effect for SAB-related late complications, like OAI in neutropenic patients, but the hazard ratio was only 0.39 (95 % CI: 0.11–1.39), and the neutropenic subgroup was relatively small ( n= 102) (Camp et al., 2022). In our study, the patients with active cancer ( n= 121) included both neutropenic and non-neutropenic patients and those with hematologic, as well as solid, tumors, and so our hypothesis cannot be directly tested due to low numbers in each subgroups.

One relevant finding was the protective association between ICU stay and OAI, observed in both univariate and multivariate analyses. This effect appeared to be independent of the slightly higher proportion of hospital-acquired bacteremia among ICU patients (42.5 %) and independent of the duration of bacteremia (2.3 d). In these patients that needed ICU admission after their positive blood cultures, the frequency of pneumonia (20.5 %) and CRBSI (15.8 %) was somewhat higher compared to the general population. There was no difference in terms of the prevalence of MRSA strains. However, outcomes were worse: 7 d mortality was higher (22.6 %), and 90 d mortality was also significantly higher (56.2 %). Given that OAI is often a complication of SAB, the observed protective association of ICU stay and OAI should be retested and corrected for 7 d mortality to avoid bias from competing risks.

A secondary objective of the study was to compare patients with an orthopedic prosthesis who did not develop PJI during SAB to those who did. The PJI subgroup was relatively small ( n= 19), and the results should therefore be interpreted with caution. The origin of bacteremia was more frequently nosocomial in the non-PJI subgroup compared to in the PJI subgroup (25.6 % vs. 5.3 %, p= 0.06). No other relevant differences were observed between the two groups. The use of [18F]FDG PET/CT was similar in both groups, with 56.6 % and 57.9 % undergoing this imaging technique. Among patients with prosthetic joint infection (PJI), the 90 d mortality was 26.3 %, which was initially not statistically different from that of patients with non-infected prostheses. However, 1-year mortality was significantly higher in the non-PJI subgroup (53.8 % vs. 26.3 %, p= 0.044). In PJI patients, the mortality rate was highest during the perioperative and early septic phases but appeared to decrease over time. PJI patients typically undergo earlier source control, prolonged targeted therapy, and closer follow-up, which may mitigate late mortality. Accordingly, these data should not be interpreted as evidence that PJI confers lower mortality.

Our study has several limitations, including its retrospective and single-center design, which may limit generalizability. The real-life nature of our study reflects the routine clinical decision-making and diagnostic practices in a high-resource hospital setting. Selection and detection biases are possible, as [18F] FDG PET/CT imaging was not applied systematically across all patients but was instead determined by the treating physician. This may have led to underestimation of OAI in groups with less frequent imaging, such as patients with cancer and ICU patients, thereby contributing to potential misclassification.

While our cohort included a relatively large number of patients with SAB, the number of cases within individual osteo-articular infection subgroups remained limited. To enhance statistical power, we grouped different types of OAI into a single composite outcome. However, this approach did not allow us to account for potentially important clinical and pathophysiological differences between these entities.

## 5 Conclusion

In this study, OAI in the context of SAB was very frequent and present in nearly one in three patients. We identified a significant association between OAI and both a longer duration of bacteremia and CA-SAB. Active cancer seems to be a protective factor against OAI in our cohort but not against the smaller subgroups of NJSA and spondylodiscitis alone and could be explained by a detection bias. Admission to the ICU ward also appeared to be a protective factor; however, this association requires further investigation and adjustment for 7 d mortality.

Particular vigilance is warranted in patients with joint prostheses who develop SAB, as they are at elevated risk for PJI. In our cohort, 32.8 % of patients with prosthetic joints and concurrent SAB developed PJI; however, no relevant differences were observed between those with and without PJI.

Overall, SAB was associated with high mortality, with a 90 d cumulative mortality rate of 39.5 %. These findings underscore the need for prompt recognition and thorough diagnostic evaluation for both primary and secondary infection foci, such as OAI. Further studies are needed to identify specific subgroups of patients with SAB who may derive the greatest diagnostic benefit from [18F]FDG PET/CT, as well as to determine the optimal timing for its use in the diagnostic work-up.

## Data Availability

Given that these are individual patient data from a monocentric study, public access via a URL in the Supplement is therefore not possible for data protection reasons. Instead, the data can be shared upon reasonable request through appropriate channels.

## Code availability

The analyses were conducted using SPSS, a menu-driven software, and no specific syntax was developed as only standard functions described in the statistical methods were used (e.g. means and logistic regression). The SPSS database can be shared upon request via appropriate channels.
